# Nanopore-based 18S rRNA metabarcoding reveals *Hepatozoon* lineages from ectoparasites

**DOI:** 10.1017/S003118202610211X

**Published:** 2026-06

**Authors:** Richard Said Thomas, Antonella Bacigalupo, María Carolina Silva-de la Fuente, Nicol Quiroga, Adriana Santodomingo, Julian Quintero-Galvis, Claudia Marina Muñoz, Laura Vega, Nathalia Ballesteros, Nicolás Luna, Diana Carolina Hernández, Juan David Ramírez González, Martin Llewellyn, Carezza Botto-Mahan, Carlos Landaeta-Aqueveque, Sebastián Muñoz-Leal, Lucila Moreno Salas

**Affiliations:** 1 Departamento de Ciencia Animal, Facultad de Ciencias Veterinarias, Universidad de Concepción, Chillán, Chile; 2 Facultad de Medicina Veterinaria y Agronomía, Universidad de Las Américas, Santiago, Chile; 3 School of Biodiversity, One Health and Veterinary Medicine, University of Glasgow, Glasgow, UK; 4 Facultad de Ciencias, Universidad de Chile, Santiago, Chile; 5 Facultad de Ciencias Agrarias y Forestales, Escuela de Medicina Veterinaria, Universidad Católica del Maule, Talca, Chile; 6 Centro de Investigación de Estudios Avanzados del Maule (CIEAM), Vicerrectoría de Investigación y Postgrado, Universidad Católica del Maule, Talca, Chile; 7 Departamento de Ciencias, Facultad de Artes Liberales, Universidad Adolfo Ibáñez, Santiago, Chile; 8 Millenium Nucleus of Patagonian Limit of Life (LiLi), Valdivia, Chile; 9 Institute of Biotechnology, Universidad Nacional de Colombia, Bogotá, Colombia; 10 Centro de Investigaciones en Microbiología y Biotecnología, School of Sciences and Engineering, Universidad del Rosario, Bogota, Colombia; 11 Department of Microbiology, Icahn School of Medicine at Mount Sinai, New York, NY, USA; 12 Global Health and Emerging Pathogens Institute, New York, NY, USA; 13 College of Public Health, University of South Florida, Tampa, FL, USA; 14 Research Ring in Pest Insects and Climatic Change (PIC2), Universidad de Chile, Santiago, Chile; 15 Departamento de Patología y Medicina Preventiva, Facultad de Ciencias Veterinarias, Universidad de Concepción, Chillán, Chile; 16 Facultad de Ciencias Naturales y Oceanográficas, Universidad de Concepción, Concepción, Chile; 17 Centro Nacional de Investigación en Ríos, Invasiones y Sistemas (IRIS), Concepción, Chile

**Keywords:** apicomplexa, haematophagous arthropods, high-throughput sequencing, long-reads, Macronyssidae, Piroplasmida, Reduviidae, Trombiculidae

## Abstract

Inverse surveillance seeks to identify potential pathogens before diseases emerge in populations. In this study, we conducted a molecular survey of eukaryotic vector-borne pathogens (VBPs) within ectoparasite species to anticipate disease outbreaks in animals and humans. We analysed fleas, lice, mites, ticks and triatomines collected from rodents, birds, carnivores and from the environment across Chile. After extracting genomic DNA, we employed an 18S rRNA gene amplicon-based sequencing strategy using Oxford Nanopore Technologies to characterize eukaryotic VBPs. Our findings revealed a narrow taxonomic range of microorganisms, with *Hepatozoon* as a well-supported taxon. A single sequence matching the genus *Babesia* was additionally confirmed via BLAST. The bioinformatic pipeline allowed the recovery of high-quality *Hepatozoon* consensus sequences, enabling robust phylogenetic and population genetic analyses and providing the first molecular record of *Hepatozoon* in triatomines and the first genetic detection of this parasite genus in trombiculid and macronyssid mites in the Americas. Three different *Hepatozoon* lineages were detected, which clustered with others found in rodents and reptiles. Analyses of molecular variance indicate that host identity may play a more influential role than geographic region in shaping the genetic differentiation of *Hepatozoon* lineages in Chile. Our study provides new data on ectoparasite–host–parasite associations, demonstrating the utility of nanopore metabarcoding data for exploring VBPs’ diversity, thereby expanding the known arthropod associations of the *Hepatozoon* genus and suggesting the role of these ectoparasites as its potential vectors. As some *Hepatozoon* species can cause animal disease, these findings constitute an early warning for veterinarians.

## Graphical abstract

**Figure:**
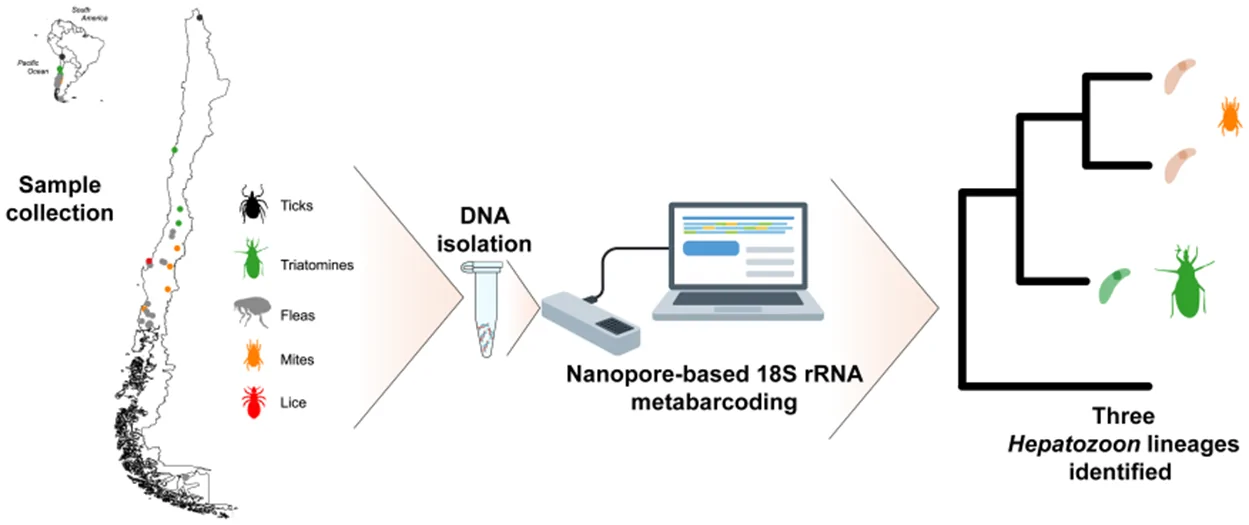

## Introduction

Ectoparasites play a key role in maintaining sylvatic cycles of eukaryotic vector-borne pathogens (VBPs, hereafter), facilitating their development and propagation across diverse ecological and geographical landscapes (Benelli and Nathan, 2019; Weitzel et al., 2020). The spectrum of eukaryotic parasites that might be transmitted by fleas (Ceratophyllidae, Hystrichopsyllidae and Pulicidae), lice (Hoplopleuridae and Haematopinidae), mites (Laelapidae), ticks (Ixodidae) and triatomines (Reduviidae) include apicomplexans such as *Babesia* spp. and *Hepatozoon* spp., as well as onchocercid filarial nematodes (e.g. *Cercopithifilaria, Cherylia, Cruorifilaria, Monanema, Yatesia* and *Acanthocheilonema*), among other VBPs (Smith, 1996; Jalovecka et al., 2019; Schnittger et al., 2022; Špitalská et al., 2022; Gruntmeir et al., 2023; Thomas et al., 2024; Ajileye et al., 2025). These parasites typically have complex life cycles involving vertebrates and ectoparasites, often resulting in health impairments and economic impact on humans, and companion and wild animals (Jalovecka et al., 2019; Schnittger et al., 2022; Gruntmeir et al., 2023; Thomas et al., 2024; Ajileye et al., 2025). Examples include babesioses in humans and animals (caused by *Babesia* spp.), while cutaneous disorders (caused by nematodes), theilerioses (caused by piroplasms) and hepatozoonosis (caused by *Hepatozoon* spp.) affect wild and domestic animals (Jalovecka et al., 2019; Schnittger et al., 2022; Gruntmeir et al., 2023; Thomas et al., 2024; Ajileye et al., 2025).

Among apicomplexans, the genus *Hepatozoon* stands out as an intriguing yet understudied group in many regions of the Americas (Thomas et al., 2024). Unlike most VBPs, which infect vertebrates via direct ectoparasite bites, transmission of *Hepatozoon* spp. occurs primarily when the hosts ingest arthropods harbouring mature *Hepatozoon* oocysts (Smith, 1996; Thomas et al., 2024). Once released in the hosts’ gut, *Hepatozoon* sporozoites migrate to target organs such as the liver, spleen and kidneys. *Hepatozoon* species exhibit a dioxenic life cycle, alternating between a wide range of vertebrate intermediate hosts (mammals, reptiles, amphibians and birds), where asexual reproduction takes place, and diverse ectoparasites (e.g. fleas, flies, lice, mites, mosquitos, ticks and triatomines), which serve as definitive hosts, where sexual maturation and reproduction occur (Smith, 1996; Telford, 2009; Baneth, 2011; Thomas et al., 2024). These vector–host associations vary by host taxon; for example, in rodents, *Hepatozoon* spp. are typically transmitted by mites, fleas and lice, whereas in lizards, triatomines can act as vectors (Smith, 1996; Telford, 2009; Thomas et al., 2024).

The molecular systematics of VBPs, including *Hepatozoon*, traditionally relies on the analysis of the small subunit ribosomal RNA (SSU, 18S rRNA) gene (Pinto et al., 2012; Jalovecka et al., 2019; Hrazdilová et al., 2020, 2021; Thomas et al., 2024). The conserved nature of this gene, interspersed with variable (V1, V3, V5, V7 and V8) and hypervariable (V2, V4 and V9) regions, allows for taxonomic resolution across diverse eukaryotic lineages (Hadziavdic et al., 2014; Jalovecka et al., 2019; Davidov et al., 2020; Reisoglu et al., 2024; Suárez et al., 2024; Thomas et al., 2024). The V4 region is highly valued in large-scale biodiversity studies due to its optimal balance of taxonomic resolution, primers with broad taxonomic coverage and strong database representation (Hadziavdic et al., 2014). In contrast, the adjacent V5 region, though shorter and less variable, complements V4 by improving amplification consistency and sequence reliability, making the V4–V5 combination a useful target for eukaryotic profiling (Hadziavdic et al., 2014).

Although high-throughput sequencing (HTS) is increasingly used for microbial surveillance and biodiversity studies, its use for exploring VBPs diversity in South America remains limited, with research focusing mainly on ticks and triatomines (Patiño et al., 2021; Martínez et al., 2023; Cruz-Saavedra et al., 2024; Ossa-López et al., 2024), and no studies are available for other ectoparasites of medical and veterinary relevance, such as fleas, lice and mites. Collecting baseline data on VBPs in these groups is the first step for detecting new pathogens before disease outbreaks occur in animals and/or people, particularly in understudied areas (Cannon et al., 2021).

Among HTS platforms, Oxford Nanopore Technologies (ONT, hereafter) offers distinct advantages, including portability, affordability, real-time data generation and the capacity to handle large sample sets with minimal infrastructure, making it well-suited for working in diverse field settings (Santos et al., 2020; Regueira-Iglesias et al., 2023; Tamames et al., 2024). Unlike Sanger sequencing, which is limited to detecting specific targets and has low throughput, or Illumina, which restricts amplicon length (typically <500 base pairs [bp, hereafter]), for efficient paired-end sequencing, ONT allows the simultaneous detection of multiple parasite lineages across many samples using longer DNA fragments (Santos et al., 2020; Patiño et al., 2021; Regueira-Iglesias et al., 2023; Cruz-Saavedra et al., 2024). In this study, we applied a nanopore amplicon-based sequencing approach targeting a long fragment of the 18S rRNA gene, using universal eukaryotic primers to explore and characterize the diversity of VBPs in Chile. To this end, we analysed fleas, lice, mites, ticks and triatomine samples collected over 10 years from the environment and from vertebrate hosts across the country.

## Materials and methods

### Collection and processing of ectoparasites

**Figure 1.**
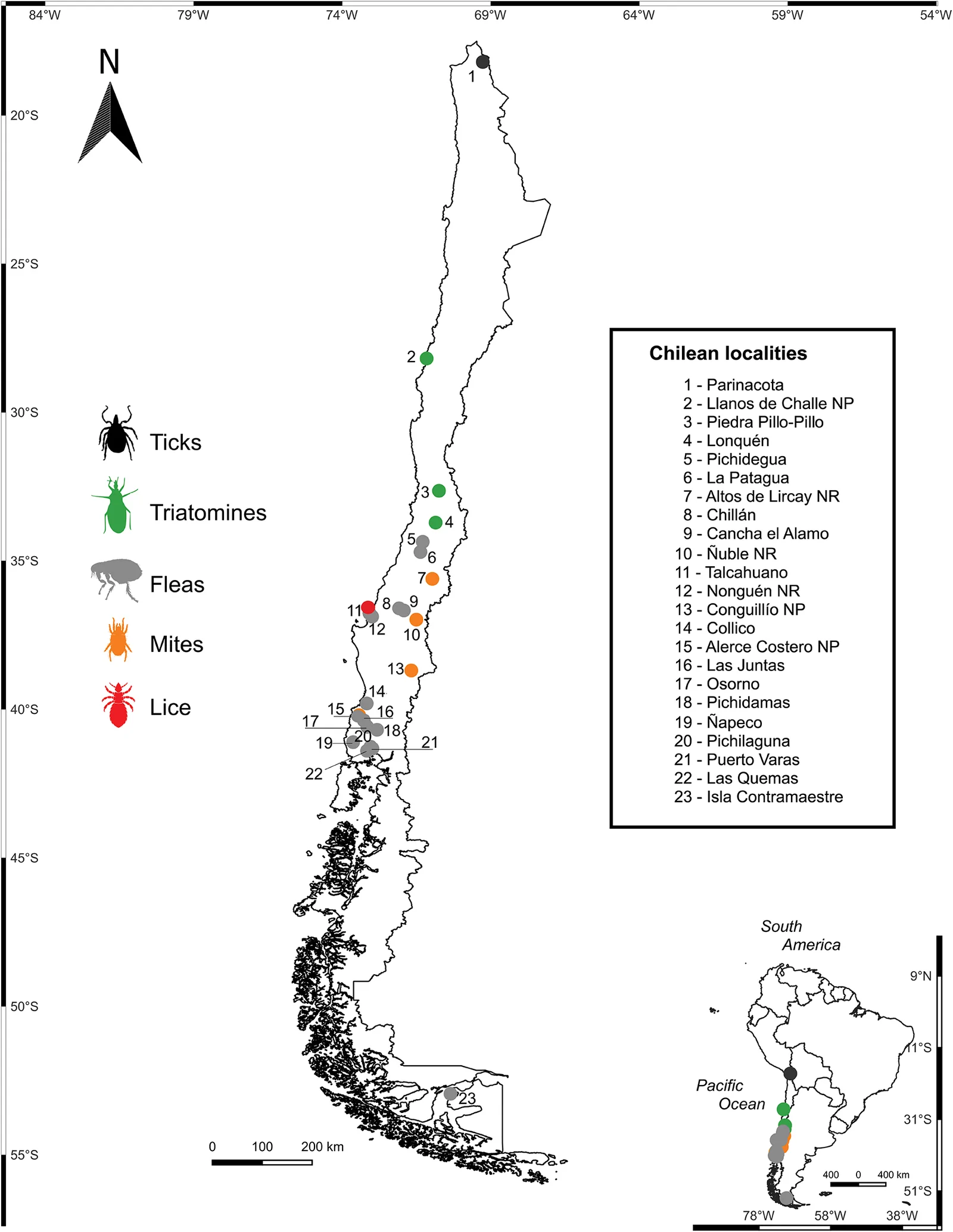
Map of Chile showing the geographic locations of ectoparasite collection sites, covering northern, central and southern regions. Abbreviations: NP, National Park; NR, National Reserve. Maps were designed with Quantum Geographic Information System (QGIS) 3.34.0-Prizren (https://www.Gnu.Org/licenses). Arthropod silhouettes were obtained from the PhyloPic portal (https://www.Phylopic.Org/) (accessed on April 4th, 2025). Figure 1 long description.

Between 2013 and 2023, 6047 ectoparasites were recovered from the environment and from 11 vertebrate host species from 23 localities across Chile (Fig. 1 and Supplementary Table S1). The hosts comprised 6 wild species from the genera *Abrothrix* and *Loxodontomys* (Rodentia: Cricetidae), *Scelorchilus* (Passeriformes: Rhinocryptidae) and *Spheniscus* (Sphenisciformes: Spheniscidae); 3 invasive species from the genera *Rattus* (Rodentia: Cricetidae) and *Neogale* (Carnivora: Mustelidae); and 1 domestic species from the genus *Felis* (Carnivora: Felidae) (Table S2). Upon collection, ectoparasites were stored at −80 °C until processing.

Ectoparasites were identified using specific taxonomic keys for fleas (Johnson, 1957), lice (Kim et al., 1986), mites (Brennan and Goff, 1977; Stekolnikov and González-Acuña, 2015; Silva-De La Fuente et al., 2021), ticks (Nava et al., 2017) and triatomines (Lent and Wygodzinsky, 1979; Frias et al., 1998; Frías-Lasserre, 2010). From the collected ectoparasites, 117 were processed individually for molecular analyses, including 43 fleas from 9 species (*Chiliopsylla allophyla, Ctenocephalides felis, Ctenoparia inopinata, Leptopsylla segnis, Neotyphloceras* sp., *Nosopsyllus fasciatus, Parapsyllus* sp., *Pulex irritans* and *Sphinctopsylla araes*), 4 lice (*Polyplax spinulosa*), 46 ticks (*Amblyomma parvitarsum*) and 24 triatomines from 3 species (*Mepraia parapatrica, Mepraia spinolai* and *Triatoma infestans*). In contrast, the 5930 mites were grouped into 43 pools according to mite species (*Derrickiella expansus, Herpetacarus* sp., *Ornithonyssus* sp. and *Quadraseta* sp.) and their associated vertebrate host species. Table S2 presents the sample origin, host species and ectoparasite taxonomic classification.

### DNA extraction

Ectoparasites were initially washed with 1% hypochlorite for 30 sec, followed by 3 washes with nuclease-free water (Promega, Madison, WI, USA) for 1 min each to eliminate external nucleic acids and surface contaminants (Lejal et al., 2020). Then, depending on the ectoparasite group, they were crushed either individually or in pools using sterile pestles as indicated previously. Genomic DNA of ectoparasites was extracted using the DNeasy Blood & Tissue Kit (QIAGEN, Hilden, Germany), following the manufacturer’s guidelines. The DNA was eluted in 40 μL buffer AE from the same kit. To account for potential contamination, we included DNA extraction blanks for each ectoparasite group, which were processed alongside the samples during washing, homogenization, DNA extraction, amplification and sequencing. DNA extraction and subsequent quality assessments for each ectoparasite group were conducted on separate days and in different laboratory rooms to prevent cross-contamination. The quantity and purity of DNA were measured using an Epoch Microplate Spectrophotometer (BioTek Instruments, Inc., Winooski, VT, USA). DNA purity was assessed using A260/A280 and A260/A230 absorbance ratios, following Khare et al. (2014) and Thermo Fisher Scientific (2009), respectively. Only samples with purity values of 1.8–2.0 for A260/A280 and 1.8–2.2 for A260/A230 were retained for further analyses.

### Nanopore sequencing

Before sequencing, DNA eluates from ectoparasites and the extraction blanks were amplified by conventional PCR (cPCR) using universal primers 566-F (5′-CAGCAGCCGCGGTAATTCC-3′) and 1289-R (5′-ACTAAGAACGGCCATGCACC-3′), which have been validated for eukaryotic DNA profiling in previous HTS studies (Hadziavdic et al., 2014; Davidov et al., 2020; Reisoglu et al., 2024; Suárez et al., 2024). These primers amplify an internal region of the 18S rRNA gene (∼723 bp), spanning from the V4 region to the beginning of the V7 region.

The suitability of primers 566-F/1289-R for this study was assessed *in silico* with the Primer-BLASTn tool (https://www.ncbi.nlm.nih.gov/tools/primer-blast/) and the SILVA TestPrime (https://www.arb-silva.de/testprime) against representative Apicomplexa and Nematoda sequences, prior to experimental amplification. After confirming their *in silico* performance, with no mismatches detected, the primers were empirically validated using *Hepatozoon canis* DNA (GenBank accession number OR814218) as a positive control. This control was used solely to confirm amplification and was not subjected to sequencing.

PCR reactions were performed on a SimpliAmp thermal cycler (Thermo Fisher Scientific, Waltham, MA). Each reaction had a final volume of 12.5 µL, consisting of 6.25 µL of LongAmp Taq 2X Master Mix (catalogue number M0287S; New England BioLabs Inc., Ipswich, MA), 1.25 µL of each primer (0.4 µM), 2.25 µL of ultrapure water and 1.5 µL of template DNA. No template controls included ultrapure nuclease-free water instead of template DNA. PCR products were purified using AMPure XP beads (Beckman Coulter) and quantified using the Qubit dsDNA High Sensitivity Assay (Life Technologies) on a Qubit 3.0 fluorometer to ensure sufficient double-stranded DNA yield for sequencing.

Purified PCR products were then used to prepare 2 independent libraries for sequencing with the SQK-LSK114 Native Barcoding Kit (ONT), following the manufacturer’s instructions. The process included end-repair and A-tailing of the PCR products using the NEBNext® Ultra^™^ II End Repair/dA-Tailing Module Kit, barcoding with the kit’s barcodes and adapter ligation using the NEBNext® Quick Ligation Module. Libraries were loaded onto 2 R10.4.1 flow cells, adding bovine serum albumin (Invitrogen^™^ UltraPure^™^ BSA 50 mg/ml) as recommended, and were sequenced for 72 h in June 2023 using 2 MinION MK1C devices running MinKNOW software v22.10.7. Seventy-three samples plus four no-template controls were loaded into 1 flow cell, while 92 samples plus 3 no-template controls were loaded into the other.

### Bioinformatic analyses

#### Data processing and quality filtering

Considering that the available bioinformatic pipelines for nanopore metabarcoding datasets, such as NanoCLust (Rodríguez-Pérez et al., 2021), NanoASV (Cousson et al., 2025) and PRONAME (Dubois et al., 2024), are optimized for full-length amplicons or reads typically exceeding 1000 bp, and the expected sequence length with the selected primers was ∼723 bp, we developed and implemented a comprehensive bioinformatics workflow using multiple open-source tools to analyse our sequencing data (Supplementary Figure S1).

FAST5 files were processed with Guppy v6.5.7, using the Super Accuracy model to ensure high-fidelity basecalling (decoding the signal to its corresponding bases), retaining only reads with a Phred quality score (*Q*-score) ≥ 10. Basecalled reads were subsequently demultiplexed using sample-specific barcodes. Only reads with barcodes at both ends were retained. The resulting FASTQ files were then concatenated by barcode, and an initial quality evaluation was conducted using NanoPlot v1.42.0 (De Coster et al., 2018). Adapters and chimeric reads were removed using Porechop v0.2.4 (https://github.com/rrwick/Porechop).

Enzyme amplification dropout, potential mutations, insertions or deletions introduced during PCR amplification, nanopore sequencing errors and natural heterogeneity in the DNA can result in variable read lengths (Acinas et al., 2005; Delahaye and Nicolas, 2021; Sultanov and Hochwagen, 2022). To address these issues, we applied length-based filtering with fastp v0.23.4 (Chen et al., 2018), retaining reads within the 577–831 bp range. This range covered the target DNA region (∼723 bp), including the hypervariable IV and variable V regions of the 18S rRNA gene (∼577 bp; Hadziavdic et al., 2014), with an additional 15% margin. This strategy ensured the inclusion of biologically relevant reads while minimizing off-target sequences and artefacts.

PCR primers were removed using Cutadapt v3.3 (Martin, 2011), which was configured to detect primers at both ends and within reads, including their reverse complements (‘--rc’ option), to account for the bidirectional nature of nanopore sequencing. Reads with primers located in the middle of the sequence were discarded. An error tolerance of 25% (−e 0.25) was applied to account for sequencing and PCR errors. The ‘--times 6’ parameter was used to iteratively remove multiple primer occurrences. After primer trimming, a second quality check was conducted using NanoPlot (De Coster et al., 2018). Quality metrics from both NanoPlot analyses were aggregated and summarized with MultiQC v1.24.1 (Ewels et al., 2016).

#### Taxonomic analysis and genetic characterization

##### Sections 2.4.2

Taxonomic classification was performed using Kraken2 v2.0.9 (Wood et al., 2019) using the non-redundant (nr) SILVA Ref NR 138.2 reference database (Quast et al., 2013) obtained from the ARB-SILVA portal (https://www.arb-silva.de/browser/) (Figure S1), which includes a set of 56 826 curated 18S rDNA sequences. Kraken2 assigns taxonomic labels by comparing sub-sequences (*k*-mers) of the reads to those in the reference database. The 18S rDNA dataset was indexed for Kraken2 with the parameters ‘--kmer-len 35’, ‘--minimizer-len 31’ and ‘--minimizer-spaces 6’ to balance computational efficiency and classification accuracy (Peterson et al., 2022) (for details, see Additional file: Kraken2_indexing_script.sh).

To improve the reliability of taxonomic classifications and reduce misassignments, we applied additional parameters: ‘--confidence 0.1’ and ‘--minimum-hit-groups 3’, following the Kraken2 handbook (https://github.com/DerrickWood/kraken2/wiki/Manual) and Lu et al. (2022). This confidence threshold ensures that taxonomic labels are only assigned with at least 10% confidence, while the second parameter guarantees that at least 3 independent sets of *k-mers* match the reference sequence before a taxonomic label is assigned.

Taxonomic classifications were visualized using the Pavian web application (https://fbreitwieser.shinyapps.io/pavian/), and the results were exported in tabular format (see Figure S1). Only assignments related to ectoparasite-transmitted VBPs at the family or genus level were retained, including those based on a single read. Although such taxonomic assignments are typically considered unreliable, they were included for exploratory purposes, particularly to detect potentially rare or undescribed taxa and highlight areas of possible occurrence. To further validate Kraken2 classifications, taxonomic identifications supported by <20 reads (low-read assignments, hereafter) were subjected to BLAST (https://blast.ncbi.nlm.nih.gov/Blast.cgi) comparisons against the NCBI nr nucleotide database.

We compiled BLAST alignment threshold criteria reported across multiple studies that evaluated inter- and intra-genus nucleotide variation in several eukaryotic taxa, including Apicomplexa and Nematodes (Inokuma et al., 2002; Dunthorn et al., 2012; Newell et al., 2013; Pearson, 2013; Hugerth et al., 2014; Wu et al., 2015; Rozej-Bielicka et al., 2017; Bazinet et al., 2018; Davidov et al., 2020; Squarre et al., 2020; Murillo-Solano et al., 2021; Patiño et al., 2021; Santodomingo et al., 2022a; Alkathiri et al., 2024; Cruz-Saavedra et al., 2024; Gaonkar and Campbell, 2024; Wang et al., 2024; Freitas et al., 2025). Taking these threshold criteria into account, we implemented the following cut-offs to validate low-read assignments and consensus sequences at the genus level using MEGABLAST: ≥95% identity, ≥98% query coverage, *E* values ≤0, bit scores ≥50 and bit-score differences ≥32 between the top hit (target taxon) and the best alternative hit (non-target taxon). MEGABLAST is optimized for the high-speed alignment of closely related sequences, demonstrating optimal performance when sequence identity is ≥95% (https://blast.ncbi.nlm.nih.gov/Blast.cgi). When multiple matches shared alignment statistics, we selected the top-listed hit as the matching sequence because it reflects the best overall alignment according to the algorithm’s ranking. Kraken2 unclassified reads were analysed via BLAST using the algorithm and criteria threshold outlined above (see MEGABLAST.sh script). These cut-offs and criteria were utilized for all BLAST analyses.

For downstream analyses, including infection rate (IR) estimates, read abundance and consensus sequence generation for each sample, only samples with at least 20 quality-filtered reads (*Q*-score ≥15) assigned to a target taxon and with no detection in negative controls were included. This threshold was selected to minimize random sequencing errors and ensure reliable consensus accuracy, as previous studies demonstrate that 5–50 nanopore reads (5–50X) are generally sufficient to produce stable and accurate consensus sequences, ensuring confidence in taxonomic identification (Loman et al., 2015; Radhakrishnan et al., 2019; Davidov et al., 2020; Espada et al., 2022). Besides, recent empirical evaluations of updated ONT sequencing using R10.4.1 flow cells with Kit 14 chemistry indicate that cross-sample artefacts, including barcode misassignment and/or index crosstalk, are typically low (≈0.002–0.05%) under stringent dual-end demultiplexing conditions (Goraichuk and Suarez, 2025; Kuba et al., 2025).

#### Consensus sequence generation and variant search

##### Section 2.4.3

We implemented several error correction steps during consensus sequence assembly and variant identification (Figure S2). After identifying samples with reads assigned to the genus *Hepatozoon*, we extracted the corresponding reads using the ‘awk’ utility based on their taxonomic identifiers. Only samples with ≥20 reads and *Q*-score ≥15 were used for consensus sequence assembly (Loman et al., 2015; Radhakrishnan et al., 2019; Davidov et al., 2020; Espada et al., 2022). Quality filtering was performed using NanoFilt v2.8.0 (De Coster et al., 2018), with the threshold determined based on average read quality evaluated using NanoPlot, and the results summarized in MultiQC.

Consensus sequence refinement was performed iteratively using a multi-step approach optimized for Nanopore data. High-quality reads were aligned to the *Hepatozoon* 18S rDNA reference sequence (MH615006) using Minimap2 v2.28-r1209 (Li, 2018). To ensure high-confidence alignments, we applied a mapping quality (MAPQ) filter using Samtools v1.21 (Danecek et al., 2021), retaining only reads with MAPQ ≥ 60 to exclude low-confidence mappings (Ruiz-Reche et al., 2019).

A draft consensus sequence was generated using Racon v1.50 (Vaser et al., 2017), which iteratively aligns sequencing reads to a reference while correcting base-level errors. Three rounds of refinement were performed, each using the previous consensus as a new reference to progressively improve the accuracy and reliability of the initial draft sequence. Medaka v2.0.1, a neural network-based polishing tool, was employed using the r1041_e82_400bps_sup_v5.0.0 correction model to eliminate systematic errors and produce a high-quality consensus (https://github.com/nanoporetech/medaka).

Consensus sequences were aligned using MAFFT v7.308 (https://mafft.cbrc.jp/alignment/server/index.html) with the G-INS-i strategy, as implemented by Thomas et al. (2024). To retain conserved orthologous positions and eliminate poorly aligned positions or unsupported insertions, the alignment was refined with Gblocks v0.91b (Castresana, 2000; Talavera and Castresana, 2007) using default parameters. This approach reduced the impact of non-orthologous positions and sequencing artefacts, ensuring a more reliable dataset for genetic analyses. Final sequences were then validated via BLAST comparisons, applying the threshold criteria previously described (see Section 2.4.2).

#### Variant identification

Considering the intraspecific variation in *Hepatozoon canis* 18S rDNA sequences, which includes up to 3 distinct types and 10 unique subtypes of 18S rDNA copies (Léveillé et al., 2019), we performed a genetic variant analysis using the Medaka variant module (Figure S2). Consensus sequences were aligned to the high-quality filtered FASTQ reads, applying the r1041_e82_400bps_sup_variant_v5.0.0 correction model to infer true single-nucleotide polymorphisms (SNPs) and insertions or deletions (indels). The resulting Variant Call Format files were analysed with Bcftools (Danecek et al., 2021) to identify and confirm high-confidence variants (Figure S2).

To facilitate the reproducibility of our analysis, we developed a set of *Bash* scripts specifically designed for 18S rRNA nanopore amplicon-based sequencing (<1000 bp), which are available in the open-source GitHub repository (rthomasshz/nanopore_amplicon_based_sequencing).

### Phylogenetic and population genetic analyses

#### Phylogenetic analysis

In molecular systematics, phylogenies are based on the character states of terminal taxa inferred from their sequences (Felsenstein, 1981; Rannala and Yang, 1996). While the primary focus is on in-group relationships, out-group sequences are essential for polarizing characters and testing evolutionary hypotheses (Hennig, 1965; Baum and Smith, 2013). Broader taxon sampling improves node resolution, supports character polarization and reduces long-branch attractions (Pollock et al., 2002; Grant, 2019; Santodomingo et al., 2021; Uribe et al., 2024). Based on these principles, *Hepatozoon* consensus sequences were analysed using the phylogenetic framework proposed by Thomas et al. (2024), along with ortholog sequences identified through BLAST comparisons. Multiple sequence alignments were performed following the same alignment parameters and software previously described (see Section 2.4.3).

Phylogenies were inferred using maximum likelihood (ML; Felsenstein, 1981) and Bayesian inference (BI; Rannala and Yang, 1996; Yang and Rannala, 1997) methods, following the procedures of Thomas et al. (2024). Both methods use probabilistic models to estimate DNA sequence evolution, accounting for base composition, substitution rates, rate heterogeneity among sites and selective pressures, to ensure robust phylogenetic inferences (Yang, 1996; Huelsenbeck et al., 2004; Ronquist et al., 2012; Baum and Smith, 2013; Nguyen et al., 2015). Phylogenetic trees were visualized and edited with FigTree v1.4.1 (http://tree.bio.ed.ac.uk/software/figtree/) and edited in Inkscape v1.1 (https://inkscape.org/es/). The consensus topology combining ML and BI tree outputs was constructed following Santodomingo et al. (2022a).

#### Haplotype analysis

Aiming to explore broader host-association patterns, we incorporated the new *Hepatozoon* genotypes into the haplotype framework established by Thomas et al. (2024) to evaluate the haplotype frequencies (HFs) and polymorphisms among consensus sequences at a broader scale. For this, the allelic phase was inferred using the PHASE module in DNAsp v6.12.03 (Rozas et al., 2017), which allows grouping of SNPs into distinct haplotypes. Subsequently, standard genetic diversity metrics were calculated, including the number of polymorphic sites (*S*), haplotypes (*H*), haplotype diversity (Hd), nucleotide diversity (*π*) and HFs. Gaps were excluded from the analysis, whereas invariant sites were retained. To contextualize haplotypes recovered from samples, HFs were visualized through a median-joining (MJ) network (Bandelt et al., 1999) generated with PopART v1.7 (http://popart.otago.ac.nz).

In addition, to explore fine-scale ecological and spatial patterns of *Hepatozoon* diversity in Chile, we focused on the subset of sequences obtained in this study, along with sequences previously identified from Chilean hosts (Merino et al., 2009; Muñoz-Leal et al., 2019; Alabí et al., 2021; Di Cataldo et al., 2022; Thomas et al., 2024), following the same procedures described in the previous paragraph. HFs were examined in relation to biogeographic context. An MJ haplotype network was constructed from Chilean *Hepatozoon* sequences. The haplotype network was annotated with host associations and paired with a map of Chile displaying the regional occurrence of each haplotype. Sampling sites were plotted using their geographic coordinates and grouped according to the biogeographic regionalization of Chile proposed by Morrone (2015), which delineates areas based on shared patterns of terrestrial biodiversity and assemblages of endemic species.

#### Genetic structure of *Hepatozoon* in Chile

A hierarchical analysis of molecular variance (AMOVA) was performed with ARLEQUIN v3.11 (Excoffier and Lischer, 2010), using *Hepatozoon* sequences identified from vertebrate and invertebrate hosts in Chile. To evaluate whether host association or biogeography better explains the genetic differentiation of *Hepatozoon* lineages, we grouped the sequences to test these 2 hypotheses by (1) clustering patterns derived from genetic analyses and the ecological and phylogenetic relationships of the associated *Hepatozoon* hosts and (2) the biogeographic regionalization of Chile proposed by Morrone (2015).

## Results

### Sample processing and sequencing performance

A total of 160 ectoparasite DNA samples were analysed. In total, approximately 1.52 million reads (Mr), corresponding to 1078.14 Mb, were generated from 166 samples, comprising 160 experimental samples and 6 DNA extraction blanks (Table S3). As expected, blank samples produced fewer reads (2558 reads; 0.57 Mb in total). The remaining blank did not yield any reads (see Table S3; flow cell 2, barcode76).

Initial quality checks revealed an average read length of 762 bp and a mean *Q*-score of 14. After quality filtering and data curation, 163/166 (98.19%) samples yielded ∼1.14 million high-quality reads (788.10 Mb), with an average read length of 696 bp (Table S4). Three experimental samples and 2 DNA extraction blanks rendered no reads after this process (Table S4).

### Taxonomic classification

Slightly more than 1.13 million reads (785.01 Mb) were successfully classified (Table S5), while 4494 reads (3.09 Mb) remained unclassified (Table S6). Among unclassified reads, 4199 (93.44%) could not be taxonomically assigned after stringent BLAST, while 11 (0.24%) showed significant similarity to non-target organisms (Table S7). A total of 6082 reads from 26 out of the 160 samples analysed (16.25%) were assigned to the VBPs (Table 1). Of these, 6080 (99.97%) from 25 samples were classified within the genus *Hepatozoon*, whereas a single read (0.02%) from 1 sample was affiliated with the family Babesiidae (Table 1). Reads assigned only to the Nematoda phylum rank were not retained, as they did not meet the established filtering criteria for VBP detections. The remaining classified reads were associated with ectoparasites and untargeted taxa (Tables S8 and S9). Taxonomic classification of blank sample reads did not resolve to lower taxonomic levels.

**Table 1.** Vector-borne parasite reads from ectoparasite samples with sampling and sequencing metadata Table 1 long description.

Ectoparasite group	Ectoparasite ID	Flow cell	Barcode ID	Source	Ectoparasite class	Ectoparasite family	Ectoparasite species	Ectoparasite specimens (*n*)	Vertebrate host species	Locality	Geographical coordinates (latitude, longitude)	Total reads after processing	Vector-borne parasite reads	
													*Hepatozoon* reads	Babesiidae reads
Mites	11	1	barcode03	Wild vertebrate host	Arachnida	Trombiculidae	*Derrickiella expansus*	200	*Abrothrix hirta*	Alerce costero NP	−40.194219, −73.434012	23 737	1673	–
Triatomine	MpLLCH04	1	barcode07	Environment	Insecta	Reduviidae	*Mepraia parapatrica*	1	–	Llanos de Challe NP	−28.185910, −71.158319	3232	278	–
Mites	36	1	barcode11	Wild vertebrate host	Arachnida	Trombiculidae	*Quadraseta* sp.	200	*Abrothrix hirta*	Conguillío NP	−38,691048, −71.673544	1549	6	–
Mites	25	1	barcode16	Wild vertebrate host	Arachnida	Trombiculidae	*Quadraseta* sp.	72	*Abrothrix hirta*	Conguillío NP	−38,691048, −71.673544	1545	8	–
Mites	37	1	barcode17	Wild vertebrate host	Arachnida	Trombiculidae	*Quadraseta* sp.	200	*Abrothrix hirta*	Conguillío NP	−38,691048, −71.673544	1676	2	–
Mites	14	1	barcode22	Wild vertebrate host	Arachnida	Trombiculidae	*Quadraseta* sp.	200	*Abrothrix hirta*	Alerce costero NP	−40.194219, −73.434012	5582	115	–
Mites	38	1	barcode24	Wild vertebrate host	Arachnida	Trombiculidae	*Quadraseta* sp.	200	*Abrothrix hirta*	Conguillío NP	−38,691048, −71.673544	1352	2	–
Mites	15	1	barcode28	Wild vertebrate host	Arachnida	Trombiculidae	*Derrickiella expansus*	200	*Abrothrix hirta*	Alerce costero NP	−40.194219, −73.434012	2531	1	–
Mites	4	1	barcode34	Wild vertebrate host	Arachnida	Trombiculidae	*Quadraseta* sp.	75	*Abrothrix hirta*	Alerce costero NP	−40.194219, −73.434012	18 062	5	–
Mites	16	1	barcode35	Wild vertebrate host	Arachnida	Trombiculidae	*Quadraseta* sp.	200	*Abrothrix hirta*	Alerce costero NP	−40.194219, −73.434012	8819	26	–
Triatomine	MpLLCH09	1	barcode38	Environment	Insecta	Reduviidae	*Mepraia parapatrica*	1	–	Llanos de Challe NP	−28.185910, −71.158319	10 166	232	–
Mites	41	1	barcode41	Wild vertebrate host	Arachnida	Macronyssidae	*Ornithonyssus* sp.	10	*Abrothrix hirta*	Altos de Lircay NR	−35.605677, −70.966300	6414	33	–
Mites	18	1	barcode46	Wild vertebrate host	Arachnida	Trombiculidae	*Quadraseta* sp.	122	*Abrothrix hirta*	Alerce costero NP	−40.194219, −73.434012	8823	3	–
Mites	42	1	barcode48	Wild vertebrate host	Arachnida	Trombiculidae	*Quadraseta* sp.	100	*Abrothrix hirta*	Conguillío NP	−38,691048, −71.673544	9883	42	–
Triatomine	MpLLCH11	1	barcode50	Environment	Insecta	Reduviidae	*Mepraia parapatrica*	1	–	Llanos de Challe NP	−28.185910, −71.158319	7995	1510	1
Mites	7	1	barcode52	Wild vertebrate host	Arachnida	Trombiculidae	*Quadraseta* sp.	65	*Abrothrix hirta*	Alerce costero NP	−40.194219, −73.434012	7394	5	–
Mites	8	1	barcode59	Wild vertebrate host	Arachnida	Trombiculidae	*Quadraseta* sp.	128	*Abrothrix hirta*	Alerce costero NP	−40.194219, −73.434012	30 427	142	–
Mites	44	1	barcode61	Wild vertebrate host	Arachnida	Macronyssidae	*Ornithonyssus* sp.	15	*Abrothrix hirta*	Altos de Lircay NR	−35.605677, −70.966300	6733	44	–
Flea	P3	1	barcode63	Invasive vertebrate host	Insecta	Leptopsyllidae	*Leptopsylla segnis*	1	*Rattus norvegicus*	La Patagua	−34.678225, −71.361999	10 027	1	–
Mites	9	1	barcode64	Wild vertebrate host	Arachnida	Trombiculidae	*Quadraseta* sp.	102	*Abrothrix hirta*	Alerce costero NP	−40.194219, −73.434012	18 124	850	–
Mites	21	1	barcode65	Wild vertebrate host	Arachnida	Trombiculidae	*Derrickiella expansus*	66	*Abrothrix hirta*	Conguillío NP	−38,691048, −71.673544	27 535	20	–
Mites	33	1	barcode66	Wild vertebrate host	Arachnida	Trombiculidae	*Derrickiella expansus*	215	*Abrothrix hirta*	Conguillío NP	−38,691048, −71.673544	8016	12	–
Mites	34	1	barcode72	Wild vertebrate host	Arachnida	Trombiculidae	*Quadraseta* sp.	127	*Abrothrix hirta*	Conguillío NP	−38,691048, −71.673544	8081	1067	–
Mites	46	2	barcode01	Wild vertebrate host	Arachnida	Macronyssidae	*Ornithonyssus* sp.	10	*Abrothrix hirta*	Altos de Lircay NR	−35.605677, −70.966300	19 321	2	–
Mites	47	2	barcode09	Wild vertebrate host	Arachnida	Macronyssidae	*Ornithonyssus* sp.	12	*Phyllotis* sp.	Altos de Lircay NR	−35.605677, −70.966300	25 290	1	–
Flea	PM171_1	2	barcode70	Wild vertebrate host	Insecta	Rhopalopsyllidae	*Parapsyllus* sp.	1	*Spheniscus magellanicus*	Isla Contramaestre	−52.941944, −70.355278	5393	–	–

Although *Hepatozoon* was detected in 25 samples, only 13 (52%) met the established inclusion criterion of ≥20 reads, qualifying them for IR and read abundance estimates (Table 1). In mites, the global IR of *Hepatozoon* was 23.26% (10/43 pools), with reads detected in 2 of 7 pools (28.57%) of *Ornithonyssus* sp., followed by *Quadraseta* sp. (6/22; 27.27%) and *D. expansus* (2/13; 15.38%). The read abundance of *Hepatozoon* also varied between mite pools, accounting for 2.76% (4012/145 335 reads) of the total reads (Table 1). Specifically, *D. expansus* harboured the highest proportion with 3.30% (1693/51 272 reads), followed by *Quadraseta* sp. with 2.77% (2242/80 916 reads) and *Ornithonyssus* sp. with 0.59% (77/13 147 reads). Triatomines, on the other hand, showed a *Hepatozoon* IR of 12.50% (3/24), with reads detected exclusively in 3 of the 7 *M. parapatrica* specimens (42.86%), resulting in a *Hepatozoon* read abundance of 9.44% (2020/21 393 reads) (Table 1).

Regarding low-read count samples (i.e. those below 20 reads), 36 of the 48 reads (76.60%) were confirmed by Kraken2 classification and BLAST analysis, with BLAST validation rates ranging from 50% to 100% across samples (Table 2). Among these, 35 (72.92%) reads assigned to the *Hepatozoon* genus showed concordance between both methods. Additionally, low read counts derived from mites matched *Hepatozoon* sequences previously characterized from *Abrothrix* rodents in Chile, whereas a single read from a flea matched a *Hepatozoon* sequence previously identified in a *Rattus* rodent (Table 2). Only a single *Hepatozoon* read, matching *H. canis* isolate Thrissur, Kerala (MG543849), did not pass BLAST validation (Table 2). On the other hand, the read initially assigned to the Babesiidae family by Kraken2 was confidently associated with the genus *Babesia* after BLAST analysis (Table 2).

**Table 2.** Summary of BLAST comparison for validation of low-read Kraken2 assignments Table 2 long description.

Ectoparasite group	Ectoparasite ID	Flow cell	Barcode	Ectoparasite species	Reads assigned to the *Hepatozoon* genus by Kraken2	Reads validated by BLAST*	BLAST validation rate (%)	Length reads (bp)	BLAST subjects	% Identity	Identities	% Query cover	*E*-value	Gaps	Bit score	Bit-score difference	GenBank accession number of matching sequences
Mites	36	1	11	*Quadraseta* sp.	6	5	83.33	713–682	*Hepatozoon* sp. isolates AO6, AO12	98.27–94.40	707–657	100–98	0	12–6	1184–1065	127 to 8	FJ719817, FJ719815
Mites	25	1	16	*Quadraseta* sp.	8	7	87.50	698–650	*Hepatozoon* sp. isolates AO6, AS15	98.56–91.65	699–626	100	0	15–5	1203–897	146 to −70	FJ719817, FJ719816
Mites	37	1	17	*Quadraseta* sp.	2	2	100	688–687	*Hepatozoon* sp. isolate AS7	97.12–96.38	694–666	100	0	10–7	1144–1118	87 to 61	FJ719819
Mites	38	1	24	*Quadraseta* sp.	2	1	50	687–679	*Hepatozoon* sp. isolate AS7	98.67–94.44	687–646	100	0	13–3	1193–1039	136 to −18	FJ719819
Mites	15	1	28	*Derrickiella expansus*	1	0	0	701	*Hepatozoon* sp. isolate AO6	93.02	653	97	0	25	1010	−47	FJ719817
Mites	4	1	34	*Quadraseta* sp.	5	3	60	694–683	*Hepatozoon* sp. isolate AO6	98.85–95.85	698–639	100–96	0	11–2	1207–1051	150 to −6	FJ719817
Mites	18	1	46	*Quadraseta* sp.	3	2	66.67	694–640	*Hepatozoon* sp. isolate AO6	98.55–90.91	696–630	100	0	55–4	1194–994	137 to −63	FJ719817
Triatomine	MpLLCH11	1	50	*Mepraia parapatrica*	1	1	100	651	*Babesia microti* isolate GXS6	95.2	634	100	0	17	1037	57	OR117717
Mites	7	1	52	*Quadraseta* sp.	4	3	75	690–613	*Hepatozoon canis* isolate Thrissur *Hepatozoon* sp. isolate AS7	99.27–90.32	694–401	100–72	1e–158 to 0	10–1	1140–573	146 to −484	MG543849, FJ719819
Flea	P3	1	63	*Leptopsylla segnis*	1	1	100	693	*Hepatozoon* sp. isolate RT9	97.4	673	100	0	4	1165	108	MT919389
Mites	33	1	66	*Derrickiella expansus*	12	9	75	690–677	*Hepatozoon* sp. isolate AS7	98.99–93.60	695–643	100	0	20–3	1211–1015	154 to −42	FJ719819
Mites	46	2	1	*Ornithonyssus* sp.	2	1	50	692–686	*Hepatozoon* sp. isolate AO6	99.42–94.06	692–649	100	0	7–1	1227–1040	170 to −17	FJ719817
Mites	47	2	9	*Ornithonyssus* sp.	1	1	100	687	*Hepatozoon* sp. isolate AO6	97.82	673	100	0	2	1167	110	FJ719817

Maximum and minimum values are provided for each BLAST metric when possible (e.g. % identity, query cover, bit score, bit-score difference). Bit-score differences were calculated using the best alternative hit (non-target taxon): *Hemolivia* sp. isolate 5060 (bit-score: 1057; GenBank accession number KF992714) for reads assigned to *Hepatozoon, Theileria* sp. HN2 (980; FJ645725) for the Babesiidae read.

* A detailed breakdown of BLAST results per individual read and sample is provided in Supplementary Table S10, where non-validated assignments are also highlighted.

### Consensus sequences, variant detection and BLAST analysis

Thirteen samples met the inclusion criterion for consensus sequence generation (≥20 *Hepatozoon*-assigned reads). After quality filters, 11 high-quality *Hepatozoon* sequences were successfully assembled. The resulting *Hepatozoon* sequences had an average length of 710 bp and a mean *Q*-score of 32 (Table S11). Following refinement with Gblocks, all consensus sequences were of 691 bp. Pairwise comparisons between the 11 *Hepatozoon* genotypes revealed 4 distinct haplotypes (Table S12). Haplotype 1 (PV776293, PV776297, PV776299 and PV776300) and haplotype 2 (PV776294, PV776295 and PV776296) consisted of identical sequences each and shared 99.86% identity between them. Haplotype 3 (PV776298 sequence) showed 99.71% identity with haplotype 1 and 99.57% with haplotype 2. Haplotype 4 (PV776301, PV776302 and PV776303) also consisted of identical sequences but diverged more strongly from the other haplotypes, showing 97.25% identity with haplotype 1, 97.10% with haplotype 2 and 99.25% with haplotype 3. BLAST validation confirmed that all consensus sequences matched host-derived *Hepatozoon* 18S rDNA sequences reported from native South American cricetid rodents and invasive geckos, with similarities ranging from 98.78% to 100% (Table 3). No variants were detected among the assembled *Hepatozoon* consensus sequences.

**Table 3.** BLAST comparisons of *Hepatozoon* consensus sequences generated from reads sequenced from ectoparasite samples Table 3 long description.

Ectoparasite group	Ectoparasite ID	Barcode Flow cell 1	Length (bp)	*Q*-score	Genotype	Accession number NCBI	Ectoparasite species	BLAST subjects	% Identity (Identities)	Query cover	*E*-value	Gaps	Bit score	Bit-score difference	GenBank accession number of matching sequences	Host origin from matching sequences	Country of origin from matching sequences
Mites	11	3	723	27	*Hepatozoon* sp. D_exAlC11	PV776293	*Derrickiella expansus*	*Hepatozoon* sp. isolate AO6	99.31 (718/723)	100	0	2/723	1279	222	FJ719817	*Abrothrix olivacea*	Chile
Triatomine	MpLLCH04	7	704	31	*Hepatozoon* sp. MpLlCH04	PV776301	*Mepraia parapatrica*	*Hepatozoon* sp. DJH-2014c isolate CHUFCL4282	99.71 (689/691)	100	0	0/691	1317	260	KM234615	*Hemidactylus mabouia*	Brazil
Mites	14	22	691	34	*Hepatozoon* sp. QuAlC14	PV776294	*Quadraseta* sp.	*Hepatozoon* sp. isolate AS7	100 (691/691)	100	0	0/691	1267	210	FJ719819	*Abrothrix sanborni*	Chile
Triatomine	MpLLCH09	38	701	32	*Hepatozoon* sp. MpLlCH09	PV776302	*Mepraia parapatrica*	*Hepatozoon* sp. DJH-2014c isolate CHUFCL4282	99.71 (689/691)	100	0	0/691	1317	260	KM234615	*Hemidactylus mabouia*	Brazil
Mites	41	41	691	47	*Hepatozoon* sp. OrAlLir41	PV776295	*Ornithonyssus* sp.	*Hepatozoon* sp. isolate AS7	100 (691/691)	100	0	0/691	1249	192	FJ719819	*Abrothrix sanborni*	Chile
Mites	42	48	691	38	*Hepatozoon* sp. QuCon42	PV776296	*Quadraseta* sp.	*Hepatozoon* sp. isolate AS7	99.86 (701/702)	100	0	0/702	1262	205	FJ719819	*Abrothrix sanborni*	Chile
Triatomine	MpLLCH11	50	735	28	*Hepatozoon* sp. MpLlCH11	PV776303	*Mepraia parapatrica*	*Hepatozoon* sp. DJH-2014c isolate CHUFCL4282	99.71 (689/691)	100	0	0/691	1317	260	KM234615	*Hemidactylus mabouia*	Brazil
Mites	8	59	691	25	*Hepatozoon* sp. QuAlC8	PV776297	*Quadraseta* sp.	*Hepatozoon* sp. isolate AO5	99.58 (706/709)	100	0	1/709	1262	205	FJ719818	*Abrothrix olivacea*	Chile
Mites	44	61	691	36	*Hepatozoon* sp. OrAlLir44	PV776298	*Ornithonyssus* sp.	*Hepatozoon* sp. isolate AO5	99.57 (689/692)	100	0	0/692	1235	178	FJ719818	*Abrothrix olivacea*	Chile
Mites	9	64	691	29	*Hepatozoon* sp. QuAlC9	PV776299	*Quadraseta* sp.	*Hepatozoon* sp. isolate AO5	99.86 (721/722)	100	0	0/722	1298	241	FJ719818	*Abrothrix olivacea*	Chile
Mites	34	72	691	29	*Hepatozoon* sp. QuCon34	PV776300	*Quadraseta* sp.	*Hepatozoon* sp. isolate AO5	99.59 (723/726)	100	0	2/726	1294	237	FJ719818	*Abrothrix olivacea*	Chile

### Genetic analyses

#### Evolutionary relationships of *Hepatozoon* lineages

**Figure 2.**
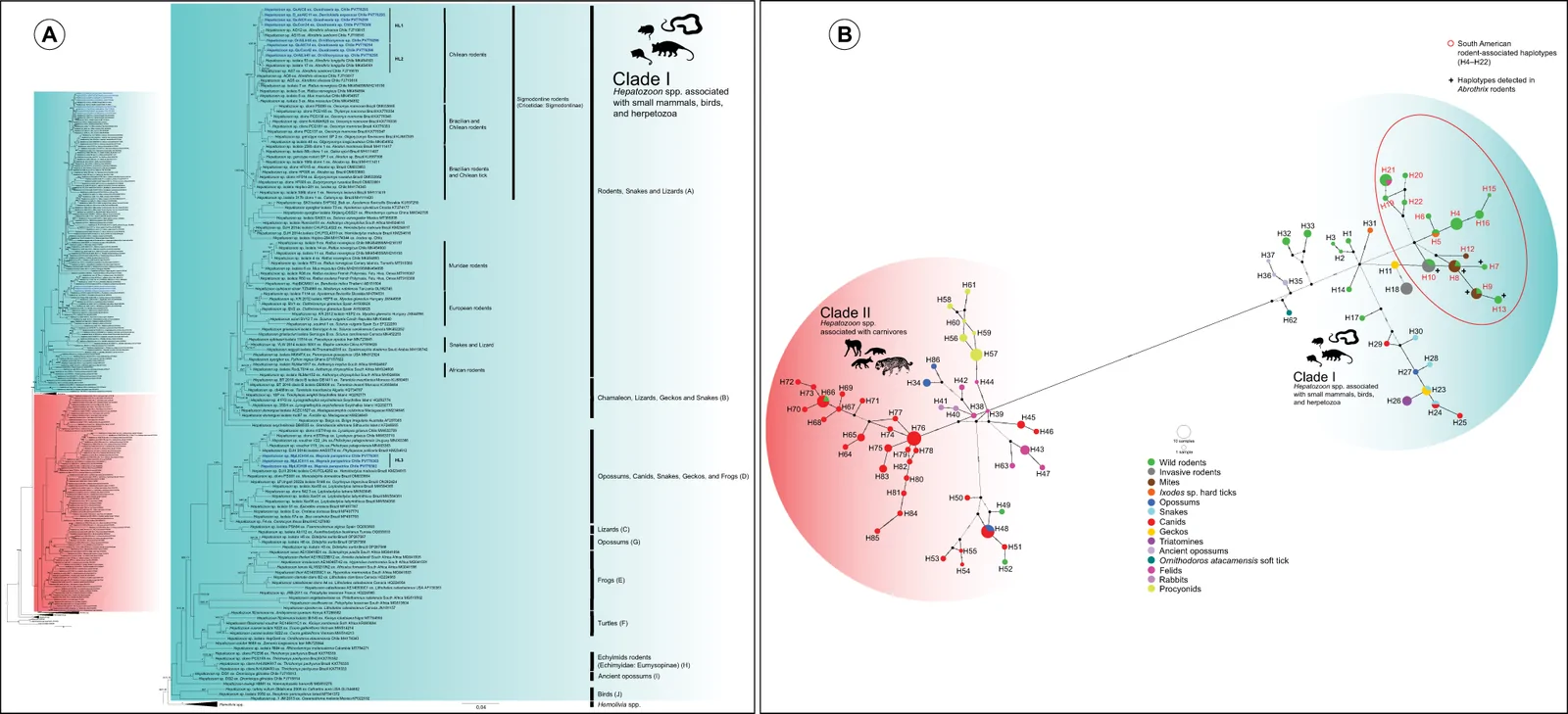
Phylogenetic and genetic analysis. Panel (A) displays the evolutionary relationships of *Hepatozoon* spp. Associated with small mammals, birds, amphibians and reptiles (Clade I), highlighting the phylogenetic placement of the *Hepatozoon* sequences characterized in this study (labelled in navy blue). HL1 and HL2 correspond to closely related subclades within a rodent-associated *Hepatozoon* complex from Chile, whereas HL3 represents a distinct lineage detected in triatomines. A consensus phylogram was inferred using maximum likelihood (ML) and Bayesian inference (BI) from 270 18S rDNA sequences of *Hepatozoon* and related haemogregarines, spanning 1985 bp. The best-fit evolutionary models calculated were TVM + F + G4 for ML and *M*_134_, *M*_85_ and *M*_15_ for BI. Node support values include ultrafast bootstrap values >70% for ML (Nguyen et al., 2015) and Bayesian posterior probabilities ≥0.71 for BI (Huelsenbeck and Rannala, 2004), shown above or below each branch. Asterisks (*) indicate maximum node support for both ML and BI. The scale bar represents nucleotide substitutions per site. *Adelina dimidiata* was used as an outgroup. The inset tree on the left depicts the complete *Hepatozoon* phylogeny. A dashed branch symbolizes the connection between this section and the rest of the phylogeny, which is fully illustrated in Supplementary Figure 3. Panel (B) shows the general haplotype network inferred from 143 *Hepatozoon* 18S rDNA sequences, covering a 409 bp alignment. *Hepatozoon* haplotypes H8–H9 represent *Hepatozoon* lineage HL1, while haplotype H12 to lineage HL2 and haplotype H23 to lineage HL3. *Hepatozoon* haplotype details are provided in Table S13. Silhouettes were obtained from the PhyloPic portal (https://www.Phylopic.Org/) (accessed on April 4th, 2025). Figure 2 long description.

Phylogenies for ML and BI trees revealed 2 major clades: Clade I predominantly associated with small mammals, birds, amphibians and reptiles (Fig. 2A), and Clade II primarily linked to carnivores (Figure S3). Likewise, the general haplotype network mirrored this phylogenetic structure, revealing a clear correspondence between haplogroups and the 2 major clades (Fig. 2B and Figure S3). Sequences obtained from mites and triatomines clustered exclusively within Clade I. Mite-derived sequences grouped closely with *Hepatozoon* lineages infecting South American rodents, alongside *Hepatozoon* sequences from *Abrothrix* rodents in Chile (Fig. 2A and B; Table S13). Similarly, *Hepatozoon* associated with triatomines fell within Clade I, forming an independent branch adjacent to *Hepatozoon* lineages related to both endothermic and ectothermic hosts across South America (Fig. 2A and B).

#### Haplotype diversity and network structure

**Figure 3.**
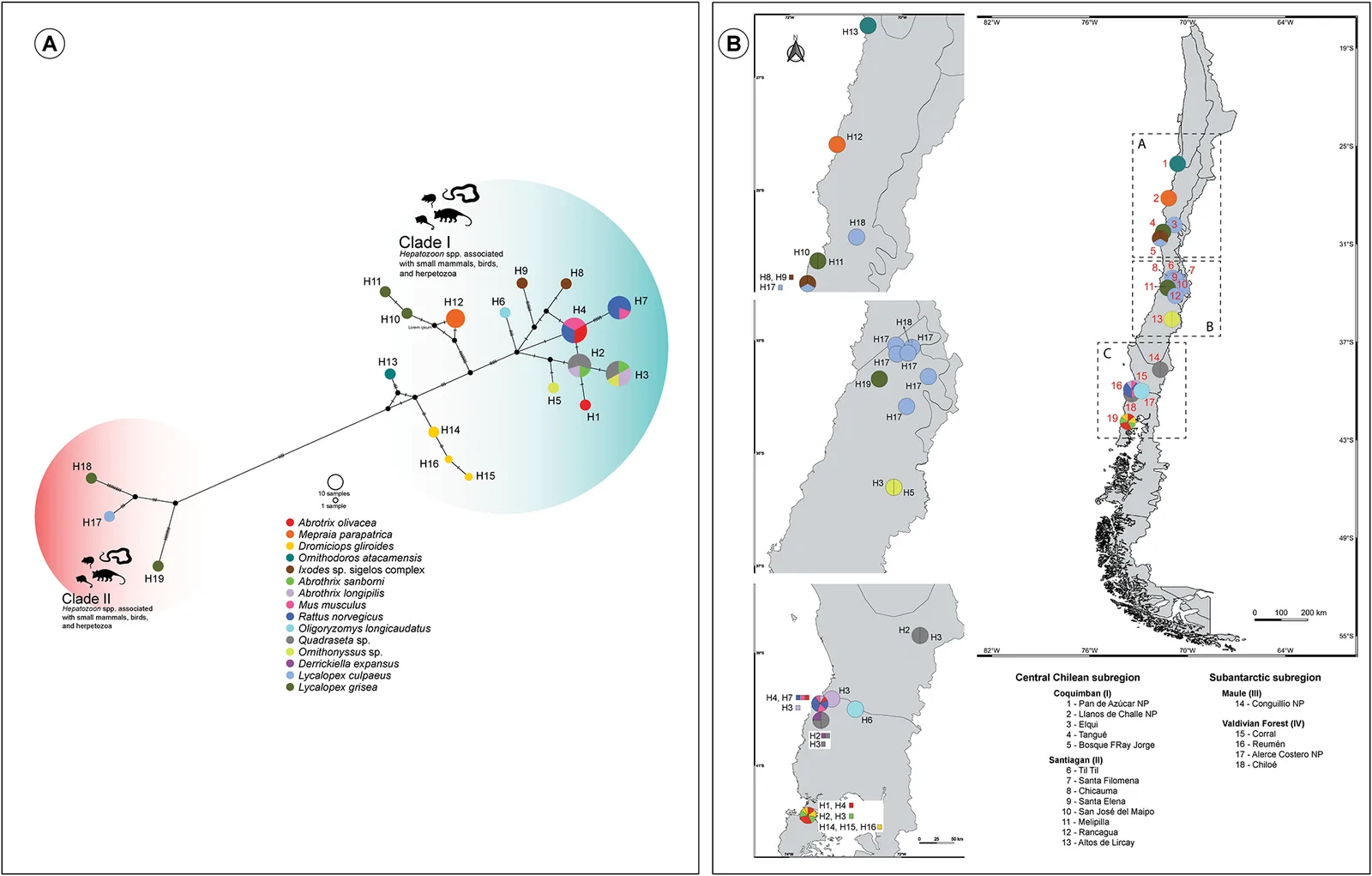
Genetic diversity and ecological patterns of *Hepatozoon* in Chile. Panel (A) shows a local haplotype network inferred from 38 *Hepatozoon* 18S rDNA sequences, covering 383 bp alignment. Panel (B) displays a map with the geographic distribution of *Hepatozoon* haplotypes across Chile, mapped using their geographic coordinates and grouped according to the biogeographic regionalization proposed by Morrone (2015). Maps were designed with Quantum Geographic Information System (QGIS) 3.34.0-Prizren (https://www.Gnu.Org/licenses). See Supplementary Tables S14 and S15 for details of the left and right panels, respectively. Figure 3 long description.

Broader polymorphism analysis of *Hepatozoon* 18S rDNA sequences, including sequences generated in this study and those previously published, yielded 86 haplotypes (Fig. 2A and Table S13), Hd = 0.983 ± 0.002, *π* = 0.05025 and *S* = 138. In contrast, local polymorphism analyses produced 19 haplotypes (Fig. 3A and Table S14), with a Hd = 0.913 ± 0.014, *π* = 0.02773 and *S* = 64. Specifically, the local haplotype network revealed at least 6 distinct haplogroups (Fig. 3A). Haplotypes H17–H19, associated with native foxes, formed 2 groups within Clade II. The remaining haplotypes were grouped into 4 haplogroups within Clade I (Fig. 3A). Haplotypes H14–H16 formed a group linked to the marsupial *Dromiciops gliroides* (Microbiotheria: Microbiotheriidae), situated adjacent to the haplotype H13, derived from a soft tick (*Ornithodoros atacamensis*).

Nearby, the haplotypes H10–H12 formed a haplogroup composed of haplotypes recovered from foxes and triatomines; however, the overall haplotype network suggested a connection to *Hepatozoon* lineages linked with reptiles (Fig. 2A and B). The haplotype from *O. atacamensis* was also grouped with reptile-associated *Hepatozoon* spp. in the phylogenetic tree (Fig. 2A), although it formed an independent group in the haplotype network. The largest haplogroup consisted of haplotypes H1–H9, primarily associated with both native and invasive rodents (Fig. 3B). Within this haplogroup, we identified 3 well-defined subclusters of closely related haplotypes (H1–H3, H4–H7 and H–H6). Interestingly, haplotypes from Clade I were widely distributed across multiple ecoregions, while Clade II was restricted to the central and northern regions (Fig. 3B).

#### Genetic differentiation of *Hepatozoon* in Chile

The first comparison in the AMOVA was conducted using 5 groups of biologically meaningful host-associated categories based on genetic analyses: (1) native rodents, (2) invasive rodents, (3) foxes, (4) marsupials and (5) reptiles. The latter group included haplotypes recovered from foxes, triatomines and 1 soft tick (*O. atacamensis*), which clustered closely with *Hepatozoon* lineages previously reported in squamate hosts (Fig. 2A and B). The analysis revealed 12.8% of genetic variation among groups of *Hepatozoon*, which was statistically significant (*F*_CT_ = 0.12840; *P* < 0.01564). In addition, variation among populations within host groups accounted for an even greater proportion of the total variance: 21.50% (*F*_SC_ = 0.24671; *P* < 0.01662) (Table S16).

On the other hand, the second model tested was performed by grouping each *Hepatozoon* haplotype by locality into the 4 biogeographic regions corresponding to Morrone’s provinces (Fig. 3B): Coquimban, Santiagan, Maule and Valdivian Forest. The AMOVA analysis indicated that Morrone’s ecoregional boundaries have a low percentage of genetic variation (0.21%). In contrast, the genetic variation among populations within groups was high: 36.12% (*F*_SC_ = 0.36194; *P* < 0.00000) (Table S16).

## Discussion

In this study, we applied ONT sequencing to survey VBPs’ diversity within a broad taxonomic scope of ectoparasites, revealing mostly *Hepatozoon* lineages, with novel host–parasite associations. Despite the increasing accessibility of HTS technologies, the application of these tools to characterize VBPs’ diversity in South American ecosystems remains limited. Nowadays, most regional efforts have concentrated on specific ectoparasite groups (particularly ticks and triatomines) to investigate vector-borne viruses (Martínez et al., 2023), vector-borne bacteria (Ossa-López et al., 2024) or specific VBPs (Patiño et al., 2021; Cruz-Saavedra et al., 2024). In contrast, other ectoparasites (such as fleas, lice and mites) and eukaryotic VBPs are absent from HTS-based studies, despite their ecological and epidemiological relevance. Our findings demonstrated that long-read metabarcoding using ONT sequencing can help bridge this knowledge gap by enabling lineage-level resolution from mixed-template samples.

Only a small proportion of reads (0.5%; 6082 reads/1.13 Mr) were assigned to VBPs (i.e. our target organisms), highlighting the challenge of detecting low-abundance taxa in complex, arthropod-derived metagenomic samples. Although our exploratory survey included a broad range of ectoparasites, *Hepatozoon* DNA was confidently detected in only 3 mite species (*D. expansus, Quadraseta* sp. and *Ornithonyssus* sp.) and 1 triatomine species (*M. parapatrica*) (Table 1). A few additional samples showed low-read assignments to *Hepatozoon* and *Babesia*, but these did not meet the confidence threshold for consensus sequence generation, and some were not supported by BLAST validation (Table 2).

Our inability to detect other VBPs (e.g. *Theileria, Cytauxzoon* and onchocercid filarial nematodes), along with low-read counts in some samples, may stem from multiple factors. These include the possible absence of these parasites in the surveyed samples; low parasite loads; dominance of host DNA; high number of samples loaded per sequencing cell; seasonal variations in IRs; limited representation of local parasite diversity in reference databases; and the use of pan-eukaryotic primers, all likely contributing to reduced detection sensitivity (Botto-Mahan et al., 2015; AL-Mayah and Abdul-Karim, 2020; Thomas et al., 2022; Kipp et al., 2023a, 2023b).

Among these factors, the use of pan-eukaryotic primers likely had a strong influence, as it led to a predominance of host DNA, limiting the relative yield of parasite reads (∼0.5% of total sequences). Nevertheless, these primers were selected to capture a wide phylogenetic range of known and potentially undescribed VBPs. While this strategy was valuable for exploratory purposes, the dominance of host DNA likely reduced the amount of parasite DNA rather than precluding parasite detection and limiting taxonomic assignments. Future efforts should focus on primer optimization, including the use of host-blocking primers, and on the application of nanopore adaptive sampling to improve recovery of target DNA while limiting multiplexing per flow cell to maximize per-sample sequencing depth. These approaches could improve taxonomic resolution and detection sensitivity (Kipp et al., 2023a, 2023b; De Meulenaere et al., 2024; Gaonkar and Campbell, 2024).

Detecting *Hepatozoon* DNA in ectoparasites does not definitively confirm their role as biological vectors of this parasite (Giannelli et al., 2013; Thomas et al., 2024). This detection could simply reflect residual, undigested blood from a recent meal, rather than an active infection in the ectoparasite. However, mites and triatomines have been implicated as vectors and definitive hosts in the epidemiology of *Hepatozoon* spp. infecting rodents and reptiles previously (Smith, 1996; Thomas et al., 2024).

Based on genetic analyses and ecological evidence, we identified 3 *Hepatozoon* lineages and 4 haplotypes from 11 consensus sequences (Fig. 2A and B). A *Hepatozoon* lineage represents evolutionary trajectories from a current species to their ancestors, defined by their genetic sequences and often shaped by genetic pressures (e.g. mutation, genetic drift or natural selection) and ecological factors – such as host use dynamics (i.e. host switching, which may lead to specialization), regional isolation, vector ecology and host community structure – reflecting specific adaptations of *Hepatozoon* spp. to different hosts, and in some cases, potentially representing undescribed or cryptic species (Léveillé et al., 2020; Hrazdilová et al., 2021; Thomas et al., 2024).

### *Hepatozoon* in mites

Phylogenetic analysis of *Hepatozoon* sequences from mites parasitizing *A. hirta* in central-southern Chile recovered 2 distinct *Hepatozoon* (HL1 and HL2) and 3 haplotypes (H8, H9 and H12) (Fig. 2A and B). Both lineages correspond to *Hepatozoon* genotypes previously detected in *Abrothrix* rodents from southern Chile (Merino et al., 2009; Alabí et al., 2021), thus representing the first detection of these rodent-associated lineages in ectoparasitic mites.

HL1 includes 5 mite-derived sequences that cluster with *Hepatozoon* sequences from *Abrothrix olivacea* and *Abrothrix sanborni* rodents on Chiloé Island, forming a subclade within Clade I (Fig. 2A and B; see Table S14 for Chilean localities). Despite minor sequence divergence among them (Table S12), these sequences were grouped into 2 haplotypes (H8 and H12), suggesting limited genetic structuring within a cohesive lineage. In contrast, HL2 comprises 3 mite-derived sequences (haplotype H9) that share ∼99.9% similarity with HL1 but form a sister subclade together with *Hepatozoon* sequences previously reported from *A. sanborni* and *Abrothrix longipilis* (Fig. 2A and B) sampled in Chiloé, Corral and Valdivia (Alabí et al., 2021), respectively (Table S14), suggesting subtle genetic structuring associated with host species or geographic origin within a rodent-associated *Hepatozoon* complex. As the mitochondrial *cox1* gene shows higher nucleotide divergence than the 18S rRNA gene, adding *cox1* sequences in future analyses could provide finer resolution to help determine whether HL1 and HL2 are distinct lineages or variants within the same *Hepatozoon* species complex (Léveillé et al., 2020; Hrazdilová et al., 2021; Thomas et al., 2024).

Trombiculid mites, such as *D. expansus* and *Quadraseta* sp., have a complex life cycle consisting of 6 free-living stages and a single parasitic larval stage (Shatrov and Kudryashova, 2006). In contrast, the macronyssid *Ornithonysus* spp. are obligate haematophagous mites throughout all life stages (Radovsky, 2010; Yin et al., 2021). Although both groups feed on a variety of small mammals, rodents appear to be their preferred host (Shatrov and Kudryashova, 2006; Yin et al., 2021). In addition, considering mites are regarded as the primary vector of *Hepatozoon* species associated with rodents (Smith, 1996; Thomas et al., 2024), the *Hepatozoon*-positive mite species identified here may be considered putative vector candidates and promising models for investigating this parasite’s persistence throughout mites’ life cycles. Nonetheless, experimental studies will be necessary to confirm these mite species as true vectors of *Hepatozoon*.

Given the mites’ ecological preference for rodents, and the fact that all *Hepatozoon*-positive mites were recovered from *A. hirta* rodents, along with the genetic similarity between *Hepatozoon* sequences found in mites and those previously reported in *Abrothrix* rodents in Chile (Fig. 2A and B), it is reasonable to assume the sympatric circulation of closely related yet distinct *Hepatozoon* lineages among *Abrothrix* species in Chilean ecosystems, likely maintained through rodent-mite interactions. Until now, only mites from the families Haemogamasidae (*Euhaemogamasus ambulans*) and Laelapidae (*Echinolaelaps echidninus*) have been reported as vectors of *Hepatozoon* spp. in the Americas, associated with North American rodents (Watkins et al., 2006). However, that investigation did not include genetic data to confirm the identity of *Hepatozoon* from mites. In contrast, our study provides the first genetic evidence of *Hepatozoon* DNA in mites of the families Trombiculidae and Macronyssidae parasitizing *A. hirta* rodents, expanding the known range of potential vectors and hosts for this parasite.

### *Hepatozoon* in triatomines

Molecular screening revealed the presence of *Hepatozoon* DNA in *M. parapatrica* from northern Chile, marking the first genetic detection of *Hepatozoon* in triatomines worldwide. Phylogenetic analyses showed that the *Hepatozoon* lineage detected in *M. parapatrica* is genetically associated with *Hepatozoon* lineages previously identified in ectothermic and endothermic hosts (Fig. 2A). Interestingly, the putative HL3 lineage clustered closely with one identified in geckos in Brazil, which also shared a haplotype with lineages found in snakes in Uruguay (Fig. 2B and Table S13).

Since *M. parapatrica* feeds on rodents, reptiles and birds (San Juan et al., 2023), the presence of *Hepatozoon* DNA likely reflects residual DNA from a previous blood meal. However, the close phylogenetic relationship of HL3 with lineages found in reptilian hosts, combined with evidence of geckos in *M. parapatrica*’s feeding profile (Quiroga et al., 2022; San Juan et al., 2023), suggests its potential involvement in a complex transmission cycle of reptile-associated *Hepatozoon* in northern Chile, beyond acting as a passive carrier. Nevertheless, this supposition requires experimental confirmation.

Although triatomines have been identified as vectors of lizard-associated *Hepatozoon* species (Smith, 1996), such evidence was based solely on morphology and precedes the molecular era, underscoring the need for an integrative systematic approach in future studies. Our results provide the first genetic evidence that triatomines may be involved in transmitting *Hepatozoon* in South America. In Chile, only *M. spinolai* was previously identified as a vector of *Hepatozoon triatomae* based on morphological evidence (Aguilera et al., 1986). Unfortunately, the absence of molecular data in that study prevents direct comparison. Notably, no *Hepatozoon* DNA was detected in *M. spinolai* specimens examined in this study. This absence of the parasite in the tested specimens may reflect a sampling effect influenced by spatial or temporal variations in infection patterns, so the potential presence of *Hepatozoon* in the sampled *M. spinolai* populations cannot be discarded. It is important to consider that the *M. spinolai* specimens analysed by Aguilera et al. (1986) were collected from Flor del Valle, which is located about 213 km north of Piedra de Pillo Pillo, where the *M. spinolai* individuals included in this study were sampled. This geographic separation might explain variations in host–parasite interactions or parasite distribution across regions.

### Taxonomic assignments supported by few reads

Despite the improved accuracy of R10.4.1 flow cells and Q20+ chemistry (https://nanoporetech.com/), residual base-calling and substitution errors, as well as multiplexing artefacts (e.g. barcode misassignment and/or index crosstalk), cannot be ruled out, meaning uncertainty remains for low- or single-read assignments. Since such classifications inherently carry a higher risk of misclassification due to limited sequencing depth and reduced taxonomic resolution (Loman et al., 2015; Radhakrishnan et al., 2019; Espada et al., 2022), they should be viewed as exploratory signals rather than definitive results.

In this study, the application of dual-end demultiplexing, together with a dual-validation taxonomic approach combining strict Kraken2 classification and confirmatory BLAST analyses, improved confidence in low-abundance signals (≤20 reads), allowing their identification at least at the genus level (Table 2 and Table S10). Even so, the absence of independent cPCR validation due to complete use of the gDNA samples during experimental procedures prevents full confirmation, emphasizing that these findings reflect trends rather than conclusive evidence. This limitation constrains the taxonomic resolution but does not invalidate the potential detection signal. Furthermore, as discussed below, the biological and ecological context of these detections provide additional support for their plausibility. Nevertheless, caution is warranted when interpreting these assignments, as definitive confirmation remains pending.

Low-read *Hepatozoon* detections in mites closely matched *Hepatozoon* sequences previously identified from *Abrothrix* rodents in Chilean ecosystems, consistent with the mites being collected while parasitizing a rodent species of the same genus (Table 2). In contrast, the single *Hepatozoon* read obtained from a *Leptopsylla segnis* flea (Table 2) resembled a *Hepatozoon* sequence found in *Rattus andamiensis* from China (Xue et al., 2024), suggesting a genus-level match consistent with the flea’s origin from an *R. norvegicus* host. Notably, *Hepatozoon* 18S rDNA sequences have shown over 99% similarity across geographically distant *Rattus* populations (Hrazdilová et al., 2021), limiting their resolution for distinguishing closely related species. These detections, along with findings of Alabí et al. (2021) and Santodomingo et al. (2022b), suggest the coexistence of distinct *Hepatozoon* lineages in Chilean ecosystems, with some associated with native rodents and others with invasive rodent species.

The single *Babesia* read from a triatomine (Table 2) was similar to a *Babesia* sequence from a *R. andamiensis* in China, associated with the *Babesia microti*-like group (Xue et al., 2024). Detecting *Babesia* in triatomines was unexpected, as only hard ticks (Ixodidae) are recognized as biological vectors of the *B. microti*-like group (Jalovecka et al., 2019; Schnittger et al., 2022). This unusual detection might be explained by rodents being part of the host profile of *M. parapatrica* in northern Chile (Quiroga et al., 2022). Since rodents are known reservoir hosts for *B. microti*-like babesias (Jalovecka et al., 2019; Schnittger et al., 2022), and the *Babesia*-positive triatomine was collected in Llanos de Challe National Park, where a *Babesia*-related lineage was previously identified in a *Phyllotis darwini* rodent (Santodomingo et al., 2022b), the detected *Babesia* DNA may derive from remnants of an undigested blood meal from an infected rodent in the triatomine’s gut. Although the detection is based on a single read, the growing evidence of *B. microti*-like diversity in South America (Santodomingo et al., 2022b, 2025; Alvez et al., 2025) suggests that such parasites may be more widespread and ecologically established than previously recognized, a possibility that merits targeted validation.

Low- or single-read assignments lacking consensus support (e.g. the *Babesia* hit and *Hepatozoon* hits lacking consensus) should be viewed only as exploratory signals rather than confirmatory findings. However, at the genus level, they still offer valuable preliminary insights into the potential presence of undescribed taxa, rare organisms and their potential ecological distributions. Retaining these signals can help illuminate target organisms, providing a basis for hypothesis generation and guiding future research, particularly in understudied ecosystems. Ultimately, these detections suggest the potential existence of multiple sylvatic cycles involving distinct *Hepatozoon* and *Babesia* lineages in Chilean ecosystems. Their precise identification, host associations with both final and intermediate hosts, and distribution patterns still need to be validated to support the biological relevance of these initial observations.

### Genetic diversity of *Hepatozoon* in Chile and ecological patterns

This study offers valuable insights into the genetic structure of *Hepatozoon* in Chile, although several limitations must be acknowledged. The 18S rRNA gene, while widely used and standardized for biodiversity studies (Hadziavdic et al., 2014; Davidov et al., 2020; Cruz-Saavedra et al., 2024; Reisoglu et al., 2024; Suárez et al., 2024), has known drawbacks, including low resolution for closely related lineages, limited ability to resolve fine-scale population structure due to its conserved and multicopy nature and potential non-neutral evolution (Rooney, 2004; Léveillé et al., 2019, 2020; Hrazdilová et al., 2021). Despite these constraints, it remains the most accessible genetic reference for *Hepatozoon* (Thomas et al., 2024). The findings presented here should thus be viewed as a baseline for comparative studies and long-term research on *Hepatozoon* in Chile. Furthermore, the observed patterns of genetic differentiation, though statistically supported, should be interpreted cautiously and considered preliminary until additional information (e.g. full-length 18S gene, mitochondrial and apicoplast markers, as well as whole genome data) is available to refine lineage boundaries.

The detection of 64 segregating sites within the Chilean dataset, compared to 138 at the continental scale, reflects substantial nucleotide variation despite the smaller geographic and taxonomic scope. This suggests the presence of multiple *Hepatozoon* lineages circulating among local hosts, consistent with previous reports of novel haplotypes in Chilean rodents, marsupials, foxes and ticks (Merino et al., 2009; Muñoz-Leal et al., 2019; Alabí et al., 2021; Di Cataldo et al., 2022; Santodomingo et al., 2022b). Both phylogenetic (Fig. 2A) and haplotype network analyses (Figs. 2B and 3A) showed a host-associated organization of *Hepatozoon* haplotypes, likely shaped by vector–host associations and host–parasite compatibility (Thomas et al., 2024). Likewise, the distinction of multiple haplogroups across both clades (Fig. 3 left) further supports non-random structuring of *Hepatozoon* lineages, consistent with patterns of host–parasite specificity observed in other studies in Chile (Merino et al., 2009; Muñoz-Leal et al., 2019; Alabí et al., 2021; Di Cataldo et al., 2022; Santodomingo et al., 2022b).

Notably, rodent-associated haplotypes formed the largest and most structured group, with 3 subclusters, suggesting that rodents can act as central host reservoirs for diverse *Hepatozoon* lineages. Their wide ecological ubiquity, high population density and interactions with multiple vectors likely facilitate parasite maintenance and spillovers (Han et al., 2020). Host-associated clustering was further supported by the AMOVA, which revealed statistically significant genetic differentiation among 5 biologically defined host categories (Table S16). Additionally, a higher proportion of genetic variation was observed among populations within host groups. These results suggest that host identity, including host population-level, may contribute to the genetic structuring of *Hepatozoon* in Chile, underscoring the central role of host biology in shaping parasite structure.

Such host-associated differentiation, in addition to that observed in Chilean rodents, has also been reported in foxes across central Chile, where *Hepatozoon* lineages appear to segregate by fox species (Di Cataldo et al., 2022). Nevertheless, the detection of similar haplotypes in distinct host taxa (e.g. foxes and triatomines, as well as native and invasive rodents) (Fig. 3A) indicates that ecological interactions, such as habitat overlap, trophic links or shared vectors, could still contribute to occasional cross-species transmission (Thomas et al., 2024). Unlike findings from Brazil, where *Hepatozoon* haplotypes from rodents showed geographic structuring (Perles et al., 2019), our AMOVA results indicated minimal differentiation across Chilean ecoregions. This is evidenced by widespread distribution of identical haplotypes across multiple localities and ecoregions (Fig. 3B), a pattern which, together with the AMOVA findings, suggests that the evaluated ecoregional barriers may be less influential in shaping *Hepatozoon* populations in Chile. The observed trends in diversity, phylogeny and population structure point to a dynamic and heterogeneous *Hepatozoon* system, where both specialist and generalist lineages coexist. Ultimately, our findings reinforce the notion that Chile harbours uniquely structured *Hepatozoon* lineages shaped by endemic hosts, highlighting the central role of host–parasite interactions in parasite evolution.

## Conclusions/future directions

This study underscores the use of nanopore amplicon-based metabarcoding to detect diversity of VBPs in ectoparasites, even from samples with low depth data. The bioinformatic workflow developed here produced high-quality consensus sequences, enabling phylogenetic and population-level analyses beyond mere taxonomic detection. Integrating ecological and biological context further strengthens the interpretation of atypical findings, allowing for more nuanced insights into parasite–host interactions. Our study identified 3 *Hepatozoon* lineages, including 2 closely related subclades (HL1 and HL2) from mites within a rodent-associated *Hepatozoon* complex and 1 distinct lineage (HL3) detected in triatomines. These results point to previously unrecognized ectoparasite-vertebrate host associations in Chile, involving mites and triatomines linked to rodents and reptile hosts, respectively. The observed haplotype diversity and phylogenetic structure suggest host-driven differentiation rather than geographic partitioning. These findings highlight complex sylvatic transmission cycles and identify neglected ectoparasite groups as potential vectors of *Hepatozoon* in Chile.

## Supporting information

10.1017/S003118202610211X.sm001 Thomas et al. supplementary material Thomas et al. supplementary material

## Data Availability

The datasets generated and/or analysed during the current study are available in the manuscript as supplementary information files. Generated sequencing files and sequences are accessible in open-access databases: FASTQ files are deposited in the Sequence Read Archive (SRA) database (https://www.ncbi.nlm.nih.gov/sra) under BioProject PRJNA1276110; FASTQ files for individual samples with *Hepatozoon* low-read counts are accessible on the Mendeley data repository (https://data.mendeley.com/) under the DOI: https://doi.org/10.17632/95ymwxcj9v.1; and GenBank accession numbers for *Hepatozoon* consensus sequences generated in this study are available at the National Center for Biotechnology Information (NCBI) nuccore database (https://www.ncbi.nlm.nih.gov/nuccore) as follows: mites (PV776293-PV776300) and triatomines (PV776301-PV776303).

## Supplementary material

The supplementary material for this article can be found at https://doi.org/10.1017/S003118202610211X.

## Figure 1 Long description

Geographic map of Chile displaying ectoparasite sampling locations collected between 2013 and 2023. Sites span multiple latitudes and include Llanos de Challe National Park, Altos de Lircay National Reserve, Conguillío National Park, and Alerce Costero National Park. Symbols denote collection points for fleas, lice, mites, ticks, and triatomines from rodents, birds, carnivores, and environmental settings. Geographic coordinates allow precise localization along Chile’s latitudinal gradient.

Navigate back to Figure 1.

## Figure 2 Long description

Panel A: Consensus phylogram inferred via maximum likelihood and Bayesian Inference from 270 Hepatozoon 18S rDNA sequences (1,985 bp). Highlights Clade I associated with small mammals, birds, amphibians, and reptiles. Novel sequences from this study are in navy blue, identifying three lineages (HL1, HL2, HL3). Support values: ultrafast bootstrap (>70%) and posterior probabilities (≥0.71). Panel B: Median-joining haplotype network of 143 sequences (409 bp). H8-H9 represent HL1, H12 represents HL2, and H23 represents HL3. Host silhouettes indicate vertebrate associations.

Navigate back to Figure 2.

## Figure 3 Long description

Panel A: Median-joining network of 38 Chilean Hepatozoon sequences (383 bp), revealing six haplogroups: H1-H9 (native and invasive rodents, largest group with three subclusters); H10-H12 (foxes and triatomines); H13 (soft tick Ornithodoros atacamensis); H14-H16 (marsupial Dromiciops gliroides); H17-H19 (native foxes). Panel B: Haplotype distribution map aligned with Morrone’s biogeographic provinces (Coquimban, Santiagan, Maule, Valdivian). Clade I haplotypes span multiple ecoregions; Clade II is restricted to central-northern areas.

Navigate back to Figure 3.

## Table 1 Long description

Metadata table for 160 ectoparasite sequencing samples. Columns: ectoparasite group, specimen ID, flow cell/barcode, source (wild host or environment), taxonomic classification (class, family, species), specimen count, vertebrate host species, locality, coordinates, total processed reads, and Hepatozoon/Babesiidae read counts. Key findings: Hepatozoon detected in 25 samples across three mite species (Derrickiella expansus, Quadraseta sp., Ornithonyssus sp.) and one triatomine (Mepraia parapatrica). Infection rates: 23.26% in mites, 12.50% in triatomines. One Babesiidae read found in a triatomine.

Navigate back to Table 1.

## Table 2 Long description

Table validating low-read (<20) taxonomic assignments via BLAST. Columns: ectoparasite group/ID, flow cell/barcode, species, Kraken2 read count, BLAST-validated reads, validation rate (%), read length, BLAST subject, alignment metrics (% identity, query cover, E-value, gaps, bit score), bit score difference vs. best alternative hit, and GenBank accessions. Results: 36/48 Hepatozoon assignments (76.60%) confirmed by BLAST; validation rates 50–100%. Mite reads matched Hepatozoon from Chilean Abrothrix rodents; one flea read matched Hepatozoon from Chinese Rattus. One triatomine Babesia read aligned with Babesia microti-like group.

Navigate back to Table 2.

## Table 3 Long description

Table presenting BLAST analysis of 11 Hepatozoon consensus sequences (691 bp post-Gblocks refinement) from mite and triatomine samples. Columns: ectoparasite group/ID, barcode/flow cell, sequence length, Q-score, genotype name, NCBI accession (PV776293–PV776303), ectoparasite species, top BLAST match, alignment statistics (99.31–100% identity, 100% query cover, E-value 0), bit scores/differences, matching sequence accessions, and host/country origin. All sequences matched Hepatozoon from South American rodents (Abrothrix spp., Chile) or geckos (Hemidactylus mabouia, Brazil), confirming four distinct haplotypes with pairwise.

Navigate back to Table 3.
