# Supplementary material for: Nanopore-based 18S rRNA metabarcoding reveals Hepatozoon lineages from ectoparasites
Source: Parasitology. 2026 May 7;153(7):861–79. doi: 10.1017/S003118202610211X (PMC13735563; doi:10.1017/S003118202610211X)
Supplement: Supplementary file 1 [file S003118202610211Xsup001.zip › Supplementary Figure legends.docx]

**Supplementary Figure legends**

**Figure S1**. Bioinformatics pipeline for Nanopore amplicon-based sequencing analysis. The process begins with basecalling and demultiplexing. The green dashed box highlights the source file (.fast5), while green arrows represent input files, and blue arrows indicate output files. Orange boxes are output files from one step and input for subsequent steps. Green dashed lines denote the reuse of generated files as inputs in subsequent steps. Blue boxes enclose the final output files.

**Figure S2**. Workflow for consensus sequence generation and genetic variant identification for *Hepatozoon* reads obtained through Nanopore amplicon-based sequencing. The green arrows represent input files, while the blue line after each box indicates output files. Each box outlines the specific tools and input/output file formats used at each step, illustrating the logical and modular structure of the process.

**Figure S3**. Maximum likelihood and Bayesian Inference tree of *Hepatozoon* spp. Major clades are indicated by background colour: Clade I (blue), which includes *Hepatozoon* spp. associated with small mammals, birds, and herpetofauna; and Clade II (red), comprising *Hepatozoon* spp. associated with carnivores.
